# Cold Ischemia Time, Kidney Donor Profile Index, and Kidney Transplant Outcomes: A Cohort Study

**DOI:** 10.1016/j.xkme.2022.100570

**Published:** 2022-11-13

**Authors:** Erik L. Lum, Piyavadee Homkrailas, Basmah Abdalla, Gabriel M. Danovitch, Suphamai Bunnapradist

**Affiliations:** 1 Kidney and Pancreas Transplant Research Center, Division of Nephrology, Department of Medicine, David Geffen School of Medicine at UCLA, Los Angeles, California; 2 Division of Nephrology, Department of Medicine, Bhumibol Adulyadej Hospital, Bangkok, Thailand

## Abstract

**Rationale & Objective:**

An average of 3,280 recovered deceased donor kidneys are discarded annually in the United States. Increased cold ischemia time is associated with an increased rate of organ decline and subsequent discard. Here we examined the effect of prolonged cold ischemia time on kidney transplant outcomes.

**Study Design:**

Retrospective observational study

**Setting & Participants:**

Recipients of deceased donor kidney transplants in the United States from 2000 to 2018.

**Exposure:**

Recipients of deceased donor kidneys were divided based on documented cold ischemia time: ≤16, 16-24, 24-32, 32-40, and >40 hours.

**Outcomes:**

The incidence of delayed graft function, primary nonfunction, and 10-year death-censored graft survival.

**Analytical Approach:**

The Kaplan-Meier method was used to generate survival curves, and the log rank test was used to compare graft survival.

**Results:**

The rate of observed delayed graft function increased with cold ischemia time (20.9%, 28.1%, 32.4%, 37.5%, and 35.8%). Primary nonfunction also showed a similar increase with cold ischemia time (0.6%, 0.9%, 1.3%, 2.1%, and 2.3%), During a median follow-up time of 4.6 years, 37,301 recipients experienced death-censored graft failure. Analysis based on kidney donor profile index (KDPI) demonstrated significant differences in 10-year death-censored graft survival, with a death-censored graft survival in recipients of a kidney with a KDPI <85% of 71.0% (95% CI, 70.5%-71.5%), 70.5% (95% CI, 69.9%-71.0%), 69.6% (95% CI, 68.7%-70.4%), 65.5% (95% CI, 63.7%-67.3%), and 67.2% (95% CI, 64.6%-69.6%), compared to 53.5% (95% CI, 51.1%-55.8%), 50.7% (95% CI, 48.3%-53.1%), 50.3% (95% CI, 46.6%-53.8%), 50.7% (95% CI, 45.1%-56.1%), and 48.3% (95% CI, 40.0%-56.1%), for recipients of a kidney with a KDPI >85%.

**Limitations:**

Heterogeneity of acceptance patterns among transplant centers, presence of confounding variables leading to acceptance of kidneys with prolonged cold ischemia times.

**Conclusions:**

Cold ischemia time was associated with an increased risk of delayed graft function and primary nonfunction. However, the effect of increased cold ischemia time is modest and has less impact than the KDPI. Transplant programs should not consider prolonged cold ischemia time alone as a predominant reason to decline an organ, especially with a KDPI <85%.

## Visual Abstract

**Figure:**
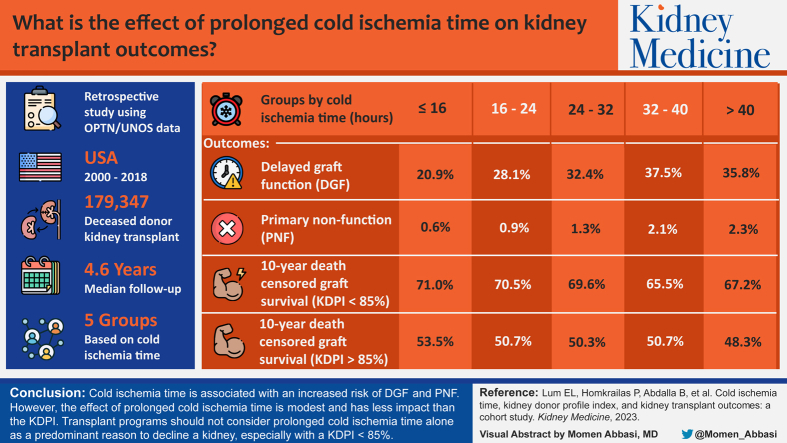

**Plain-Language Summary**

One of the main considerations in whether to accept or decline a kidney is the amount of time that has passed since the kidney has been recovered, also known as cold ischemia time. As the time the kidney has been out of the donor increases, there are concerns that it develops irreversible ischemic injury and is no longer viable. We sought to determine if very long cold times resulted in inferior kidney transplant graft survival. We examined the outcomes of kidney transplants with different cold ischemia times and found that as cold time increased, kidney transplant outcomes were worse. However, this effect was small, and more important to graft outcomes was the quality of the kidney.

Kidney transplantation is considered the optimal therapy for eligible candidates with kidney failure. Despite the clear advantages of transplantation in terms of life expectancy, quality of life, and overall health care savings, not all candidates experience its benefits. For the past 5 years, the overall number of kidney transplants has increased annually, yet there are still over 90,000 candidates awaiting transplantation. The average waiting time for a kidney transplant is 3.4 years for first-time listed candidates and 4.3 years for repeat transplant candidates and varies according to blood type and the geographic location, such that waiting time may reach more than a decade for some candidates.^1^ In 2020, it was estimated that 13 candidates died and 20 candidates were removed each day from the United Network of Organ Sharing waiting list while awaiting transplantation.^2^ Despite this, an average of 3,280 recovered deceased donor kidneys are discarded annually in the United States, which is higher than other countries.^3^

In December 2019, to achieve broader and more equitable sharing of deceased donor kidneys and minimize the impact of geography on organ allocation, the board of the Organ Procurement and Transplantation Network approved a proposal to prioritize deceased donor kidney offers for candidates listed at transplant hospitals within a 250-nautical mile radius of the donor hospital. This proposal replaced the use of local donor service areas and regions in the allocation algorithm.^4^ For candidates beyond the 250-nautical mile radius and those organs traversing donor service areas, the cold ischemia time (CIT), which is the time from organ procurement to transplantation, may be increased. Increased CIT is associated with an increased rate of organ decline by transplant centers and consequent organ discard.^5^ We analyzed the Organ Procurement and Transplantation Network/United Network of Organ Sharing database for the study period years 2000 to 2018 to examine the effect of CIT on kidney transplant outcomes.

## Methods

### Data Source and Study Population

The Organ Procurement Transplantation Network/United Network for Organ Sharing database as of September 2020 was used in this study. All deceased donor kidney transplants (DDKTs) from January 2000 to December 2018 were included. Kidneys are classified according to the kidney donor profile index (KDPI), which combines 10 donor factors to form a metric of quality for deceased donor kidneys relative to others recovered. Kidneys with KDPI ≥85% are projected to have reduced graft survival when compared to kidneys with 0%-85%.^6^

Multiple organ or double kidney DDKTs, DDKTs with missing data for the KDPI or CIT were excluded. This study was institutional review board exempt because of its use of publicly available data and absence of identification of individual donors and recipients. We grouped DDKTs based on documented CIT as follows: ≤16, 16-24, 24-32, 32-40, and >40 hours.

### Outcome Measures

The study population was analyzed to determine the graft outcomes of longer CIT kidney utilization. The primary outcome of interest was death-censored graft survival, defined as the time from transplant to the earliest of allograft loss, kidney retransplantation, reinitiation of dialysis, or loss to follow-up with a functioning graft, censored for death. Secondary outcomes included delayed graft function (DGF), defined as dialysis within the first week post-transplantation, primary nonfunction (PNF), defined as permanent loss of allograft function starting immediately after transplantation, and serum creatinine at the first year posttransplant.

### Statistical Analysis

Donor, recipient, and transplant characteristics were evaluated. Variables were analyzed using median and interquartile range for continuous variables and proportions for categorical variables. Demographic differences and posttransplant outcomes related to kidney allograft between groups were compared using Kruskal-Wallis or Pearson’s χ^2^ test as appropriate. Serum creatinine levels at the first year posttransplant were only available for individuals who were alive, being followed clinically, and did not experience graft failure at 1-year follow-up. The Kaplan-Meier method was used to generate survival curves, and the log rank test was used to compare graft survival between groups. Because donor kidney quality also affects graft outcomes, we separated survival curves into KDPI <85% and ≥85%. In regression analysis, CIT was modeled with restricted cubic splines with 4 knots located at 16, 24, 32, and 40 hours. Cox proportional hazards model was used to calculate hazard ratios (HRs) and 95% confidence intervals (CIs) to examine risks associated with graft loss. A logistic regression model was used to calculate odds ratio (OR) and 95% CI to examine risks associated with DGF and PNF. CIT of 16 hours was used as a reference. Because of a low number of DDKTs with a CIT >48 hours (0.5%), we grouped donors with a CIT ≥48 hours together in the regression analysis plots. In the multivariable model, we adjusted for (1) donor sex, smoking, cytomegalovirus status, and KDPI; (2) recipient age, sex, African American ethnicity, diabetes, cytomegalovirus status, hepatitis C virus status, and previous kidney transplant; and (3) transplant variables, which included machine perfusion pump used, panel reactive antibody, human leukocyte antigen mismatch, and induction immunosuppression. STATA version 13 (Statacorp) was used in all statistical analyses.

## Results

There were 223,429 DDKTs between January 1, 2000 and December 31, 2018. We excluded 44,082 DDKTs from the study (28,165 were multiorgan transplants, 5,529 were double kidney transplants, 9,169 had missing CIT values, and 1,219 had missing KDPI values). A total of 179,347 DDKTs were included in our analysis. Median follow-up time was 4.6 (0-17.9) years. Among these, 50,704 (28.3%) recipients died and 37,301 (20.8%) recipients experienced death-censored graft failure. DDKTs were divided into 5 groups based on documented CIT: ≤16, 16-24, 24-32, 32-40, and >40 hours, representing 83,870 (46.8%), 59,782 (33.3%), 25,017 (14.0%), 7,377 (4.1%), and 3,301 (1.8%) recipients, respectively. Baseline donor, recipient, and transplant characteristics are shown in Table 1. Longer CIT groups were more likely to have a higher KDPI, but there was no clinically significant difference of donor and recipient characteristics among groups. A significantly higher proportion of machine perfusion pumps used were observed among longer CIT groups.

**Table 1:** Baseline Donor, Recipient, and Transplant Characteristics by Cold Ischemic Time

Variables	≤16 h N = 83,870 (46.8%)	16-24 h N = 59,782 (33.3%)	24-32 h N = 25,017 (14.0%)	32-40 h N = 7,377 (4.1%)	>40 h N = 3,301 (1.8%)	*P*
Median cold ischemic time, h (IQR)	11 (8-14)	20 (18-22)	27 (26-29)	34 (35-37)	45 (42-49)	
Donor characteristics						
Median age, y (IQR)	38 (24-50)	39 (25-51)	40 (25-51)	43 (27-53)	42 (27-53)	<0.01
Female, n (%)	33,045 (39.4%)	17,456 (29.2%)	9,832 (39.3%)	(40.6%)	(41.7%)	
African American, n (%)	10,819 (12.9%)	7,652 (12.8%)	3,252 (13.0%)	(14.1%)	(15.1%)	<0.01
Smoking, n (%)	19,877 (23.7%)	15,424 (25.8%)	6,805 (27.2%)	2,110 (28.6%)	937 (28.4%)	<0.01
Hypertension, n (%)	19,876 (23.7%)	15,663 (26.2%)	6,980 (27.9%)	2,404 (32.6%)	1,806 (54.7%)	<0.01
Diabetes, n (%)	4,781 (5.7%)	3,946 (6.6%)	1,901 (7.6%)	693 (9.4%)	303 (9.2%)	<0.01
CMV positive, n (%)	51,245 (61.1%)	36,586 (61.2%)	15,786 (63.1%)	4,854 (65.8%)	2,116 (64.1%)	<0.01
HCV positive, n (%)	2,013 (2.4%)	2,033 (3.4%)	1,076 (4.3%)	243 (3.3%)	66 (2.0%)	<0.01
Median terminal serum creatinine, mg/dL (IQR)	0.9 (0.7-1.2)	1.0 (0.7-1.3)	1.0 (0.7-1.4)	1.0 (0.8-1.5)	1.1 (0.8-1.7)	<0.01
Cerebrovascular cause of death, n (%)	26,419 (31.5%)	19,369 (32.4%)	8,306 (33.2%)	6,533 (35.7%)	1149 (34.8%)	<0.01
Donation after cardiac death, n (%)	9,645 (11.5%)	9,984 (16.7%)	3,552 (14.2%)	1,099 (14.9%)	511 (15.5%)	<0.01
Median KDPI, % (IQR)	36% (15%-59%)	40% (18%-63%)	42% (19%-65%)	48% (25%-71%)	48% (25%-69%)	<0.01
Donor with KDPI ≥85%, n (%)	4,607 (5.5%)	4,553 (7.6%)	2,195 (8.8%)	830 (11.3%)	308 (9.3%)	<0.01
Recipient characteristics						
Median age, y (IQR)	52 (39-61)	53 (42-63)	53 (43-62)	55 (44-64)	55 (44-64)	<0.01
Female, n (%)	33,129 (39.5%)	23,913 (40.0%)	10,207 (40.8%)	2,848 (38.6%)	1,319 (40.0%)	<0.01
African American, n (%)	26,588 (31.7%)	19,130 (32.0%)	8,206 (32.8%)	2,552 (34.6%)	1,195 (36.2%)	<0.01
Diabetes, n (%)	24,658 (29.4%)	19,011 (31.8%)	8,031 (32.1%)	2,516 (34.1%)	1,145 (34.7%)	<0.01
Peripheral vascular disease, n (%)	5,284 (6.3%)	3,886 (6.5%)	1,526 (6.1%)	479 (6.5%)	223 (6.8%)	<0.01
CMV positive, n (%)	54,516 (65.0%)	39,815 (66.6%)	17,061 (68.2%)	5,113 (69.3%)	2,331 (70.6%)	<0.01
HCV positive, n (%)	4,194 (5.0%)	3,467 (5.8%)	1,626 (6.5%)	457 (6.2%)	139 (4.2%)	<0.01
Cause of ESRD, n (%)						<0.01
Glomerular diseases	13,168 (15.7%)	8,668 (14.5%)	3,427 (13.7%)	966 (13.1%)	389 (11.8%)	
Diabetes	20,129 (24.0%)	15,663 (26.2%)	6,654 (26.6%)	1,991 (27.0%)	921 (27.9%)	
Hypertension	19,877 (23.7%)	14,288 (23.9%)	6,204 (24.8%)	2,104 (28.5%)	963 (29.2%)	
kidney transplant, n (%)	8,806 (10.5%)	5,918 (9.9%)	2,477 (9.9%)	723 (9.8%)	287 (8.7%)	<0.01
Median dialysis vintage, d (IQR)	1,361 (743-2,157)	1,358 (754-2,151)	1,320 (727-2,095)	1,326 (747-2,027)	1,284 (742-1,956)	<0.01
Previous kidney transplant, n (%)	9,477 (11.3%)	7,891 (13.2%)	3,352 (13.4%)	869 (11.8%)	309 (9.4%)	<0.01
Transplant characteristics						
Machine perfusion pump used, n (%)	22,561 (26.9%)	25,168 (42.1%)	12,508 (50.0%)	4,845 (65.7%)	2,627 (79.6%)	<0.01
Median HLA mismatch, n (IQR)	4 (3-4)	4 (3-5)	4 (3-5)	4 (3-5)	4 (4-5)	<0.01
Median peak PRA, % (IQR)	0% (0%-28%)	0% (0%-39%)	0% (0%-40%)	0% (0%-23%)	0% (0%-14%)	<0.01
Induction immunosuppression, n (%)						<0.01
None	13,587 (16.2%)	9,804 (16.4%)	4,003 (16.0%)	996 (13.5%)	366 (11.1%)	
Antithymocyte globulin	37,825 (45.1%)	26,423 (44.2%)	10,932 (43.7%)	3,253 (44.1%)	1,349 (40.9%)	
Anti-IL-2R antibody	18,367 (21.9%)	12,434 (20.8%)	5,078 (20.3%)	1,195 (16.2%)	321 (9.7%)	
Alemtuzumab	8,556 (10.2%)	6,875 (11.5%)	2,652 (10.6%)	819 (11.1%)	347 (10.5%)	
Combined	2,348 (2.8%)	1,913 (3.2%)	1,301 (5.2%)	671 (9.1%)	788 (23.9%)	

Abbreviations: CMV, cytomegalovirus; ESRD, end-stage renal disease; HCV, hepatitis C virus; HLA, human leukocyte antigen; IL-2R, interleukin 2 receptor; IQR, interquartile range; KDPI, kidney donor profile index; PRA, panel-reactive antibody level.

### DGF

The overall incidence of DGF was stable during the study period but was higher among longer CIT groups: 17,538 (20.9%), 16,776 (28.1%), 8,101 (32.4%), 2,767 (37.5%), and 1,182 (35.8%) among CIT ≤16, 16-24, 24-32, 32-40, and >40 hours, respectively (Table 2). Compared to a CIT of 16 hours, a longer CIT was associated with a significantly increased risk for DGF (Fig S1).

**Table 2:** Kidney Transplant Outcomes

	≤16 h N = 83,870 (46.8%)	16-24 h N = 59,782 (33.3%)	24-32 h N = 25,017 (14.0%)	32-40 h N = 7,377 (4.1%)	>40 h N = 3,301 (1.8%)	*P*
Overall delayed graft function, n (%)	17,538 (20.9%)	16,776 (28.1%)	8,101 (32.4%)	2,767 (37.5%)	1,182 (35.8%)	<0.01
KDPI <85%	16,127/79,263 (20.4%)	15,176/55,229 (27.5%)	7,274/22,822 (31.9%)	2,423/6,547 (37.0%)	1,060/2,993 (35.4%)	<0.01
KDPI ≥85%	1,411/4,607 (30.6%)	1,600/4,553 (35.1%)	827/2,195 (37.7%)	344/830 (41.5%)	122/308 (39.6%)	<0.01
Overall primary non-function, n (%)	903 (1.1%)	977 (1.5%)	440 (1.8%)	183 (2.5%)	73 (2.2%)	<0.01
KDPI <85%	764/79,263 (1.0%)	728/55,229 (1.3%)	357/22,822 (1.6%)	146/6,547 (2.2%)	60/2,993 (2.0%)	<0.01
KDPI ≥85%	139/4,607 (3.0%)	149/4,553 (3.3%)	83/2,195 (3.8%)	37/830 (4.5%)	13/308 (4.2%)	0.15
Overall median serum creatinine at 1-y posttransplant, mg/dL (IQR)	1.3 (1.0-1.6)	1.3 (1.0-1.7)	1.4 (1.1-1.7)	1.4 (1.1-1.8)	1.4 (1.1-1.8)	<0.01
KDPI <85%	1.3 (1.0-1.6)	1.3 (1.1-1.7)	1.3 (1.1-1.7)	1.4 (1.1-1.8)	1.4 (1.1-1.8)	<0.01
KDPI ≥85%	1.6 (1.3-2.1)	1.7 (1.3-2.1)	1.7 (1.3-2.1)	1.7 (1.4-2.2)	1.7 (1.4-2.2)	0.06

Abbreviations; IQR, interquartile range; KDPI, kidney donor profile index.

### PNF

There was a steady decrease in the overall incidence of PNF between 2000 and 2015; however, the rate was stable in the years 2015-2018 at 0.6%, 0.9%, 1.3%, 2.1%, and 2.3% among CIT ≤16, 16-24, 24-32, 32-40, and >40 hours, respectively (Table 2, Fig S2). Compared to a CIT of 16 hours, a longer CIT was associated with a significantly increased risk for PNF (Fig S3).

### Serum Creatinine at First Year Posttransplant

There were no clinical differences in median serum creatinine at the first year after DDKT, which were 1.3 mg/dL (IQR, 1-1.6), 1.3 mg/dL (IQR, 1-1.7), 1.4 mg/dL (IQR, 1.1-1.7), 1.4 mg/dL(IQR, 1.1-1.8), and 1.4 (IQR, 1.1-1.8) mg/dL among CIT ≤16, 16-24, 24-32, 32-40, and >40 hours, respectively (Table 2).

### Long-term Graft Survival

**Figure 1:**
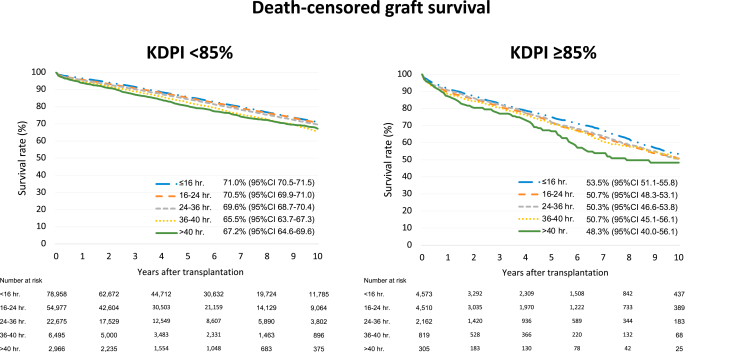
Death-censored graft survival by kidney donor profile index (KDPI).

**Figure 2:**
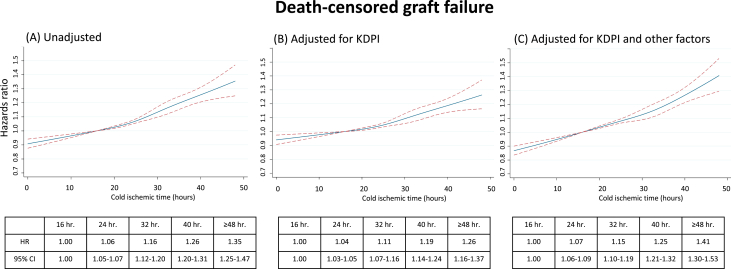
Risk of cold ischemic time (CIT) on death-censored graft failure. CIT was modeled with restricted cubic splines with 4 knots located at 16, 24, 32 and 40. Hazard ratios (HRs) were plotted against CIT, and 95% confidence intervals were included (dashed line). CIT of 16 hours was used as a reference. (A) Unadjusted HR. (B) Adjusted HR for Kidney Donor Profile Index (KDPI). (C) Adjusted HR for donor variables (sex and KDPI), recipient variables (age, ethnicity, diabetes, cytomegalovirus status, hepatitis C virus status, previous kidney transplant), and transplant variables (panel reactive antibody, human leukocyte antigen mismatch, induction immunosuppression, and machine perfusion used).

Among donors with a CIT <16, 16-24, 24-32, 32-40, and >40 hours, respectively, 16,592 (19.8%), 12,571 (21.0%), 5,533 (22.1%), 1,803 (24.4%), and 802 (24.3%) recipients experienced death-censored graft failure during the follow-up time. Ten-year overall death-censored graft survival rates were 70.1% (95% CI, 69.6-70.6), 69.1% (95% CI, 68.6-69.7), 68.1% (95% CI, 67.2-68.9), 63.9% (95% CI, 62.2-65.6), and 65.5% (95% CI, 63.1 – 67.8), respectively. There were 12,493 DDKT recipients (7%) receiving a kidney with KDPI ≥85%. When kidney quality was categorized using the KDPI, 10-year death-censored graft survival rates among CIT groups with a KDPI <85% were 71.0% (70.5-71.5), 70.5% (69.9-71.0), 69.6% (68.7-70.4), 65.5% (63.7-67.3), and 67.2% (64.6-69.6), respectively. If KDPI was ≥85%, 10-year death-censored graft survival rates were 53.5% (51.1-55.8), 50.7% (48.3-53.1), 50.3% (46.6-53.8), 50.7% (45.1-56.1), and 48.3% (40.0-56.1), respectively (Fig 1). Comparing with CIT of 16 hours, a longer CIT was associated with an increased risk for death-censored graft failure in an unadjusted Cox proportional hazards model with an HR of 1.06 (95% CI, 1.05-1.07), 1.16 (95% CI, 1.12-1.20), 1.26 (95% CI, 1.20-1.31), and 1.35 (95% CI, 1.25-1.47), respectively, among CIT of 24, 32, 40 and 48 hours (Fig 2A). After adjustment for KDPI (Fig 2B) and KDPI along with other factors as described in the methods (Fig 2C), CIT was an independent risk for graft failure with adjusted HRs of 1.07 (95% CI, 1.06-1.09), 1.15 (95% CI, 1.10-1.19), 1.25 (95% CI, 1.21-1.32), and 1.41 (95% CI, 1.30-1.53), respectively.

### Organ Discards

During the study period, United Network of Organ Sharing did not provide the specific causes of organ discard. However, these data were available from the OneLegacy Organ Procurement Organization that recovers approximately 10% of all deceased donor kidneys in the United States. Out of a total of 3,500 deceased donor kidneys that were recovered by OneLegacy between 2015 and 2019, 295 (8.7%) were ultimately discarded because of prolonged CIT alone or prolonged CIT along with other factors.

## Discussion

Increased CIT is associated with an increased rate of organ decline by transplant centers and consequent organ discard. Our study confirms the concerns surrounding increased CIT and organ transplant outcomes as DGF and PNF increased with each CIT interval studied. In a multivariate regression analysis adjusted for recipient and donor characteristics including the KDPI, a longer CIT >24 hours was associated with an increased risk for DGF, PNF, and eventual graft failure, an observation that has been previously reported.^7^, ^8^, ^9^, ^10^, ^11^, ^12^ However, our analysis revealed 2 important novel findings: (1) there was no statistically significant difference in 1-year serum creatinine and 10-year death-censored graft survival for those kidneys functioning at 1-year posttransplant; and (2) kidneys with a KDPI <85% and prolonged ischemia time, even <40 hours, performed better than kidneys transplanted with a KDPI >85% with minimal CIT. Taken together, this data suggests that CIT alone, especially in higher-quality organs, should not be the singular reason for organ decline and discard.

Serum creatinine values 1 year post kidney transplantation has been shown to be one of the best predictors for long-term kidney allograft survival. In this study, overall median serum creatinine at 1 year posttransplant did not change with increasing CIT interval (1.3 mg/dL), and a survival analysis showed comparable 10-year death-censored graft survival between CIT <16 hours and the longer CIT groups. Although CIT duration did not result in observable differences in 1 year serum creatinine, there were some key differences in each group, including higher rates of donor hypertension, donor diabetes, and terminal creatinine. One striking observable difference between the groups was the increased use of machine perfusion with increasing CIT group, with 26.9% in those with CIT <16 hours and 79.6% in those with CIT >40 hours. The effect of machine perfusion on reducing DGF/PNF and in resistance indices predicting DGF and discard have been well documented. It is possible that the use of machine perfusion provided additional information in the acceptance of organs with increased CIT or provided a milieu for limiting additional injury to the transplanted organ. Additional studies are necessary to determine the potential confounding and/or beneficial effects of machine perfusion on organs experiencing prolonged CIT.

Organ quality, as determined by KDPI, has an effect on long-term transplant survival. Higher KDPI organs, which represent lesser quality organs, are more likely to have a higher serum creatinine 1-year posttransplant and lower long-term graft survival, which our study also confirmed. CIT has a negative potentiating effect in combination with KDPI, resulting in additionally worse outcomes with higher rates of DGF and PNF. However, in those transplants that functioned, CIT interval did not correlate with adverse outcomes. As expected, our study demonstrates that CIT in high-quality organs outperformed those with a KDPI >85%. Unexpectedly, we found that those organs with a CIT >40 hours with KDPI <85% performed better than those with minimal CIT and a KDPI >85%. This is likely driven by of our finding that 1-year serum creatinine was unrelated to CIT and the much lower incidence of PNF seen in the KDPI <85% group. However, the low number of organs transplanted in this group may limit this conclusion. This suggests that lower KDPI organs can better endure ischemic injury and recover from DGF without long-term detrimental effects to long-term graft function.

One clear need arising from our results is the ability to better predict the risk for PNF, as our data clearly demonstrates that if the organ is functional, short and long-term graft survival is comparable across CIT exposure. However, the potential impact of utilizing high-quality organs with prolonged CIT should not be ignored, especially in light of recent organ allocation changes. An inevitable consequence of the goal of reduction in the geographic disparities in organ allocation is an increase in CIT, as deceased donor kidneys are more likely to be transported over longer distances and across organ procurement organizations. During the study period national data for specific reasons for discard were not provided. However, utilizing data from OneLegacy Organ Procurement Organization, which recovers approximately 10% of all deceased donor kidneys in the United States, revealed that 8.7% of kidneys were discarded for prolonged CIT alone or with other factors. Extrapolating the OneLegacy organ acceptance data highlighting the role of CIT as a contributing factor for organ decline nationally, utilizing organs with longer CITs could increase the number of overall transplants by several hundred each year.

The primary limitation of our study is the lack of information on discarded organs. It is unclear if the outcomes reported are more favorable because of careful organ selection and/or the presence of additional mitigating factors not documented in the database. The low number of patients at risk in the CIT group >40 hours in recipients with a KDPI >85% also limits conclusions for this group.

In conclusion, kidneys with prolonged CIT, which are frequently discarded, may provide years of dialysis independence for patients on the transplant waiting list. Transplant programs should not consider prolonged CIT as a predominant reason to decline an organ, a recommendation that is particularly relevant when kidneys are transported beyond the 250-nautical mile radius.
