# Supplementary material for: Cold Ischemia Time, Kidney Donor Profile Index, and Kidney Transplant Outcomes: A Cohort Study
Source: Kidney Med. 2022 Nov 13;5(1):100570. doi: 10.1016/j.xkme.2022.100570 (PMC9827060; doi:10.1016/j.xkme.2022.100570)
Supplement: Supplementary File (PDF) — Fig S1-S3. [file mmc1.pdf]

Figure S1

Delayed graft function

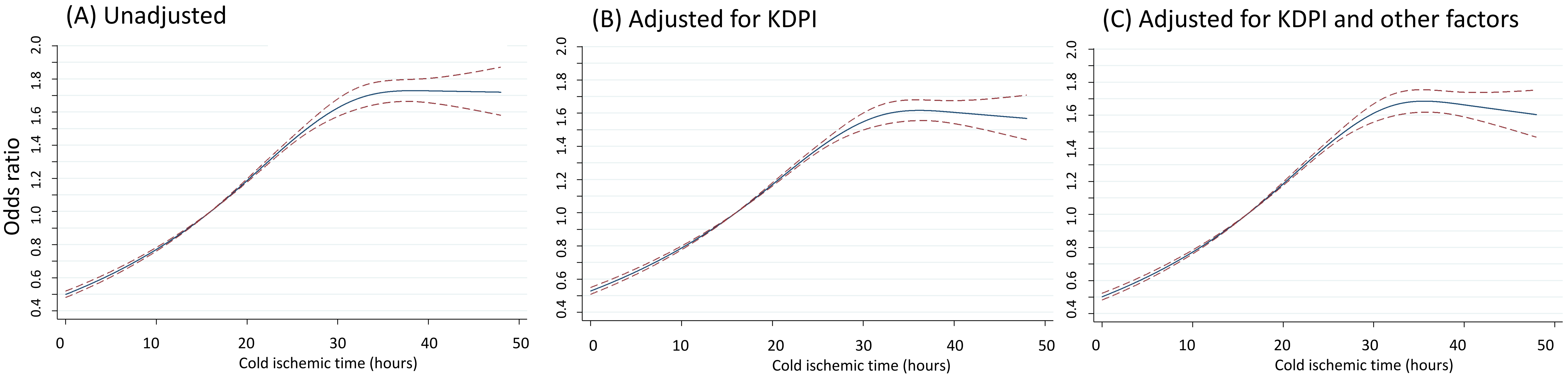

	≤16 hr.	24 hr.	32 hr.	40 hr.	≥48 hr.
OR	1.00	1.38	1.68	1.73	1.72
95% CI	1.00	1.36-1.40	1.61-1.75	1.66-1.80	1.58-1.88

≤16 hr.	24 hr.	32 hr.	40 hr.	≥48 hr.
1.00	1.35	1.59	1.60	1.57
1.00	1.32-1.36	1.53-1.65	1.54-1.68	1.44-1.70

≤16 hr.	24 hr.	32 hr.	40 hr.	≥48 hr.
1.00	1.38	1.65	1.66	1.60
1.00	1.37-1.40	1.59-1.72	1.59-1.74	1.47-1.76

Risk of cold ischemic time (CIT) on delayed graft function. CIT was modeled with restricted cubic splines with four knots located at 16, 24, 32 and 40. Odds ratio (OR) were plotted against CIT, and 95% confidence intervals were included (dash-line). CIT of 16 hours was used as a reference. (A) Unadjusted OR. (B) Adjusted OR for Kidney Donor Profile Index (KDPI). (C) Adjusted OR for donor variables (gender and KDPI), recipient variables (age, ethnicity, diabetes, cytomegalovirus status, hepatitis C virus status, previous kidney transplant) and transplant variables (panel reactive antibody, human leukocyte antigen mismatch, induction immunosuppression and machine perfusion used).

Lum et al, Kidney Med, "Cold Ischemia Time, Kidney Donor Profile Index, and Kidney Transplant Outcomes: A Cohort Study"

Figure S2

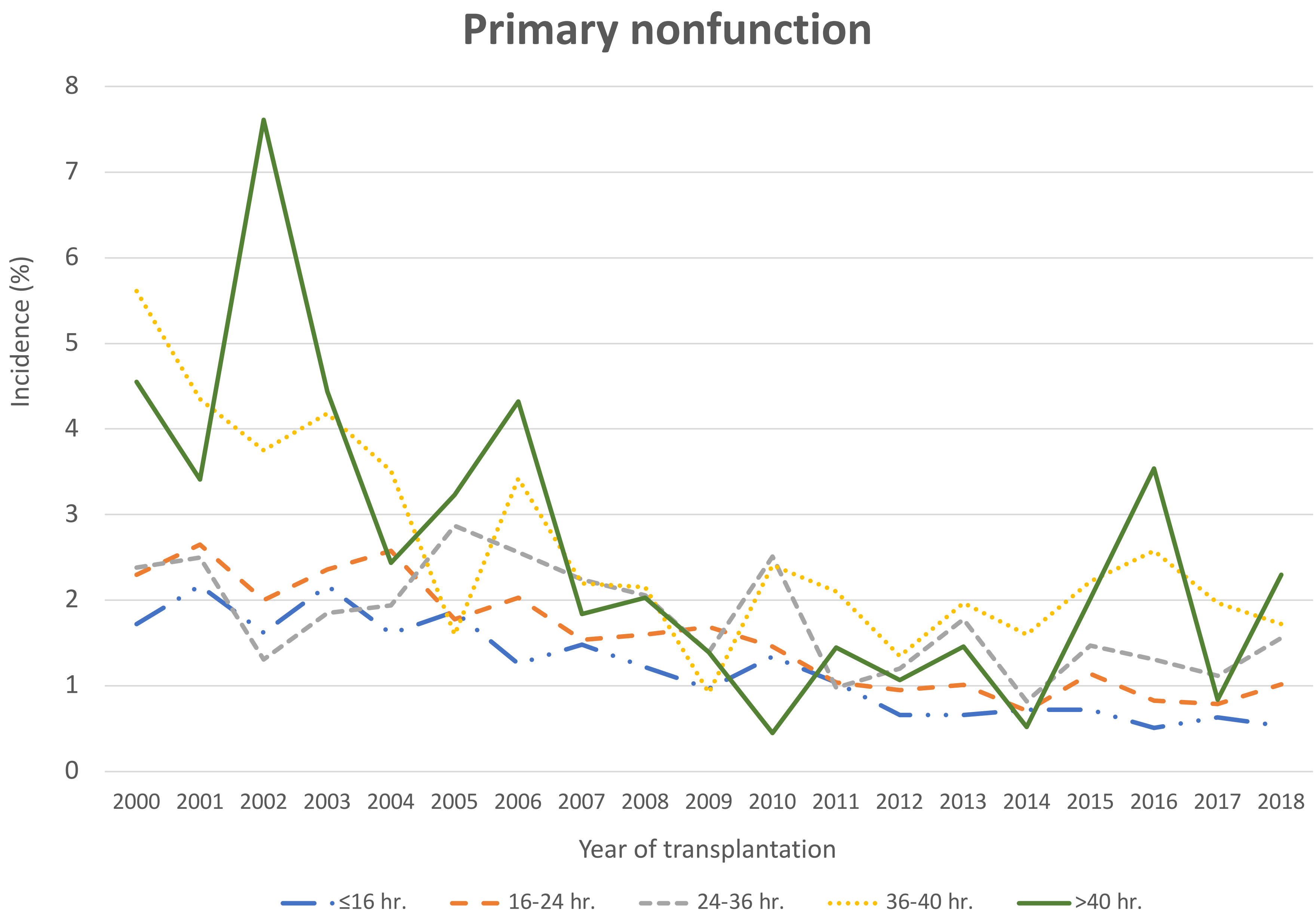

Figure S3

Primary non-function

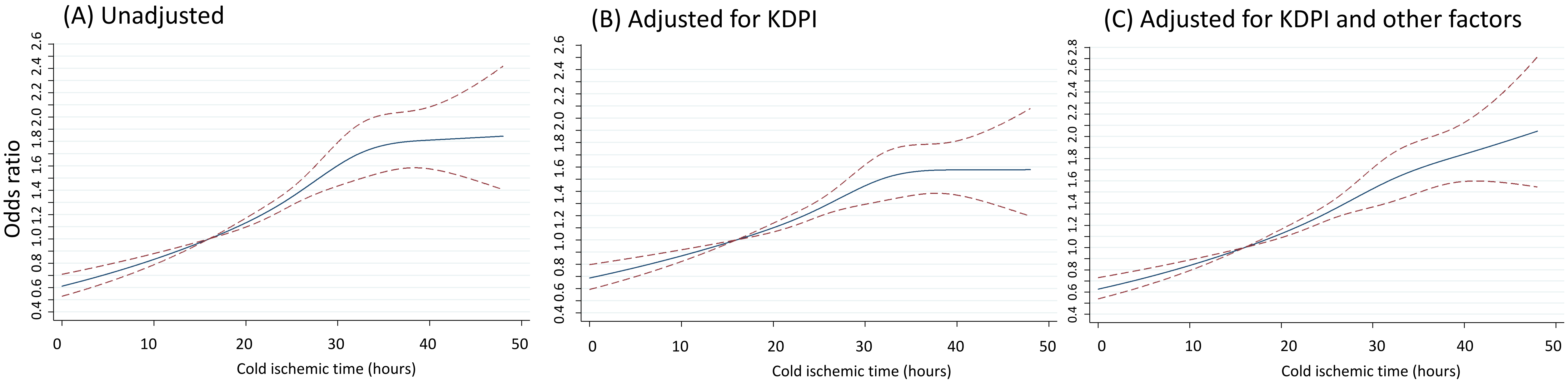

	16 hr.	24 hr.	32 hr.	40 hr.	≥48 hr.
OR	1.00	1.30	1.69	1.81	1.84
95% CI	1.00	1.24-1.35	1.49-1.92	1.58-2.08	1.40-2.42

16 hr.	24 hr.	32 hr.	40 hr.	≥48 hr.
1.00	1.22	1.50	1.57	1.58
1.00	1.16-1.28	1.32-1.71	1.37-1.72	1.20-2.08

16 hr.	24 hr.	32 hr.	40 hr.	≥48 hr.
1.00	1.27	1.60	1.84	2.05
1.00	1.21-1.33	1.40-1.84	1.69-2.12	1.44-2.71

Risk of cold ischemic time (CIT) on primary non-function. CIT was modeled with restricted cubic splines with four knots located at 16, 24, 32 and 40. Odds ratio (OR) were plotted against CIT, and 95% confidence intervals were included (dash-line). CIT of 16 hours was used as a reference. (A) Unadjusted OR. (B) Adjusted OR for Kidney Donor Profile Index (KDPI). (C) Adjusted OR for donor variables (gender and KDPI), recipient variables (age, ethnicity, diabetes, cytomegalovirus status, hepatitis C virus status, previous kidney transplant) and transplant variables (panel reactive antibody, human leukocyte antigen mismatch, induction immunosuppression and machine perfusion used).
